# Synthesis and surface characterization of new triplex polymer of Ag(I) and mixture nucleosides: cytidine and 8-bromoguanosine

**DOI:** 10.1016/j.heliyon.2019.e01609

**Published:** 2019-05-15

**Authors:** Lamia L.G. Al-mahamad

**Affiliations:** Department of Chemistry, College of Science, Mustansiriyah University, Baghdad, Iraq

**Keywords:** Inorganic chemistry, Materials chemistry, Analytical chemistry, Physical chemistry

## Abstract

In this work one-dimensional (1D) triplex polymer of silver (I): mixture nucleosides of cytidine and 8-bromoguanosine was synthesised. The polymer showed high stability due to the presence Ag(I) ions in the structure of the polymer in addition to the stability that produces from the effect of Hoogsteen hydrogen bonding in the triplex CGC. Atomic Force Microscopy (AFM) and transmission electron microscopy (TEM) were used to investigate the morphology of the polymer. The AFM images revealed formation of nanofibres extending many microns in length with height in the range of 2–3 nm. Statistical analyses carried out to analyse the AFM images to determine the height of the loops that formed in the polymer. The data displayed that the height value was in the range between 10 nm to 15 nm. The data of TEM images were consistent with the data of AFM images by displaying a very long fibre. Gwyddion software program was used to investigate surface parameters (roughness and waviness), diameter (size distribution), and probability density of the fibre. The data showed that the diameter of the fibre was ∼0.4 nm.

## 1 Introduction

**Fig. 1:**
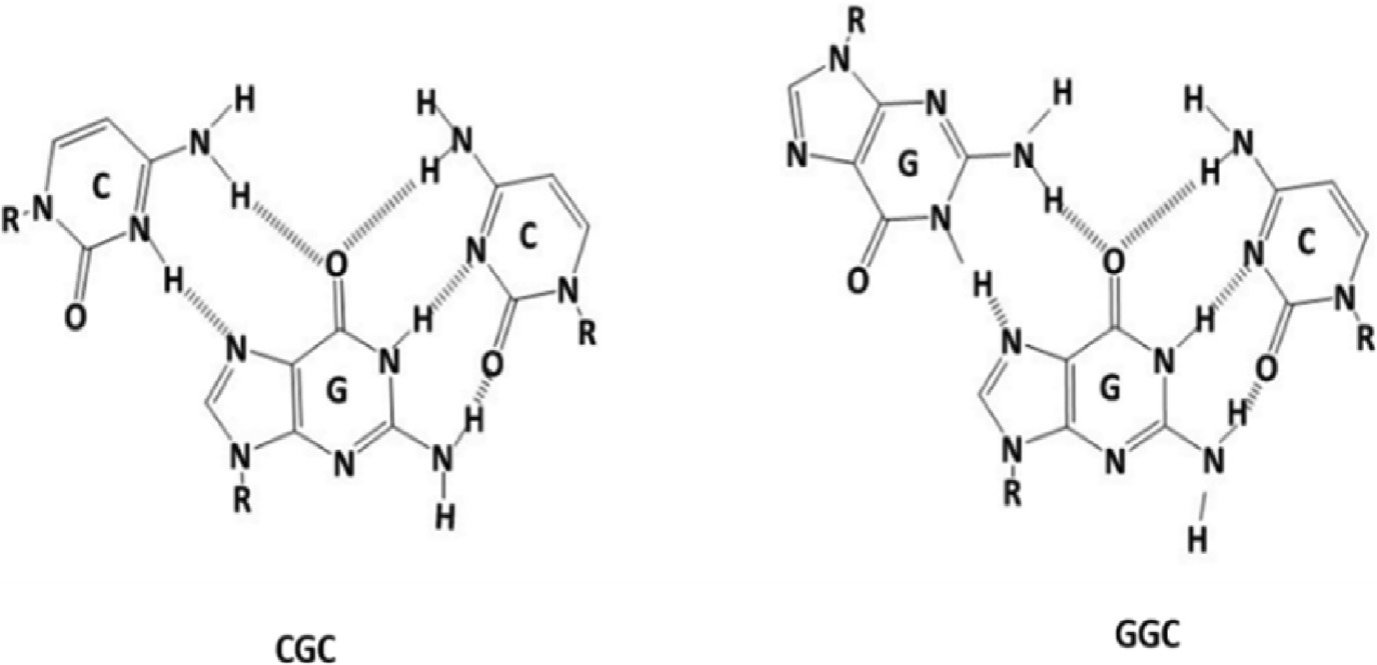
Triplexes structures of parallel motif CGC (Y pyrimidine motif) and antiparallel motif GCG (R purine motif) bases.

Based on the interactions of intermolecular hydrogen bonding, guanine and cytosine nucleobases can assemble to make long chains and forming networks, according to the known Watson-Crick pairing in both DNA and RNA [1]. Hydrogen bonding played an important role in the field of supramolecular chemistry [2] by forming different structures such as ribbons or fibres. Nucleobases and nucleosides are good examples of natural materials that depend on hydrogen bonding in their self-assembling. Another factor that helps forming larger structures in these compounds is the presence of multi binding sites in their structures [3] which increase the prone of these materials to self-assemble and produce structures of one dimension (1D), two dimensions (2D), and three dimensions (3D). Nucleobases and nucleosides showed attractive attention due to multifunctional properties of these structures such as conductivity [4], magnetism [5, 6], luminescence [7, 8], drug delivery, bioactivity, porosity [9], gas sensor, and nanotechnology [10]. Guanosine nucleoside has shown the ability to form G-quartet by self-assembling via hydrogen bonding [11]. Some of guanosine G-quartet were formed in the presence of metal ions such as K^+^, Na^+^, Ag^+^, etc. [12], while others were formed in the absence of the effect of metal ions [13]. Triplex moiety is common in the reactions that involve guanosine and cytidine. Triplex structure consists of three triple structures where the third strand is binding to a duplex Waston-Crick purine strand, when the binding of the third strand occurs in an antiparallel way by reverse Hoogsteen hydrogen bonds; then the triplex is called purine motif (R), such as GGC, AAT, and TAT. In contrast, when the binding takes place in a parallel way via Hoogsteen hydrogen bonds, then this kind of triplex motif is called pyrimidine motif (Y), e.g., CGC, [14], Fig. 1 displays structures of R and Y. Both 8-bromo guanosine [15] and cytidine [16] showed the ability to form hydrogel with Ag(I) ions, while in triplex CGC no hydrogel was reported for these nucleosides with Ag(I) ion and this probably owing to the lack in the binding sites that occurs by forming triplex CGC with Ag(I) ion.

Studying surface roughness properties of the compounds, e.g., surface of semiconductor materials, is very important to know the changing that occurs on in the surface during the chemical reaction by forming or removing a layer from the surface. The atoms in the surface possess high chemical reactivity than that in the bulk as these atoms have the ability to alter their electronic structure by reaction with different kinds of chemical environments, these properties have found applications in different industrial technologies [17]. Semiconductor materials such as silicon and GaN characterise with high electron velocity and high electron mobility, and for this reason numerous researches have been reported to study the surface of these compounds which used in different applications such as electronics, optoelectronics, and sensors [18, 19, 20, 21]. In addition, in micro fluid devices, studying how the micro/nano scale influences in surface roughness is very important to develop devices with small-scale [22]. Here, one dimensional (1D) polymer of Ag(I) with a mixture of nucleosides: cytidine and 8-brpmoguanosine was prepared. The morphology of the polymer was characterised by using AFM and TEM techniques. The surface texture of the polymer was analysed with Gwyddion software program to indicate the statistical parameters of the surface roughness and to find the probability density, in addition to the diameter of the fibres. Preparing nanomaterial compounds with a large scale to use in nanotechnology applications still represents a hard task due to the difficulty controlling the synthesis process and the self-assembling of these compounds, as these compounds can change with the surrounding environment especially their chemical and stability properties rather than bulk materials [17]. Surface chemistry has the potential approach to solve this problem by understanding the relationship between physical-chemical properties of materials and their surface topography based on AFM characterizations, statistical analysis, and surface roughness analysis to provide better insight on the useful applications of these materials.

## 2 Materials and methods

### 2.1 Chemicals and materials

All chemicals were purchased from Sigma Aldrich and were used as received without further purification. ^1^H and ^13^C NMR spectra were performed on a Bruker Advance 300 spectrometer at 300 MHz, with DMSO-d_6_.

### 2.2 Preparation silicon chips for AFM

P-silicon (100) wafers were used to achieve AFM measurements by cutting the wafers into 1 cm^2^ and cleaning with 1:4 H_2_O_2_:H_2_SO_4_ (piranha solution) for 1 h, followed by washing the chips with deionised water and drying with nitrogen gas.

### 2.3 Atomic Force Microscopy (AFM) measurements

Sample for AFM measurements was prepared by drop-casting 2 μL of the sample onto 1 cm^2^ clean silicon chip, then the sample was dried by air prior to scan with NanoScope Analysis 1.5 software (Bruker).

### 2.4 Transmission electron microscopy (TEM) measurements

TEM measurements were carried out by using Philips CM100 electron microscope at accelerating voltage 100 kV. 2 μL of the sample was dropped onto a carbon coated copper grid, the sample was left to dry by air overnight before imaging.

### 2.5 Surface texture parameters analysis

Gwyddion software program was used to analysis AFM images.

### 2.6 Synthesis of derivative nucleoside: 8-bromo guanosine

8-Bromoguanosine was prepared according to the procedure of Srivastava [23]. N-Bromosuccinimide (0.4 g, 2.24 mmol) was added to the suspension of guanosine (0.566 g, 2 mmol in 16 mL anhydrous DMF) the suspension was constantly stirred for 24 h at room temperature. The resulting clear yellow solution was concentrated under reduced pressure to remove the solvent at 50 °C. The residue was collected by adding some water, followed by filtering the solid product and recrystallized by using hot water and drying by air (colourless crystal was yield (0.707 g with respect to the weight of guanosine, 80 %).

#### 2.6.1 ^1^H NMR characterization of 8-bromoguanosine

**Fig. 2:**
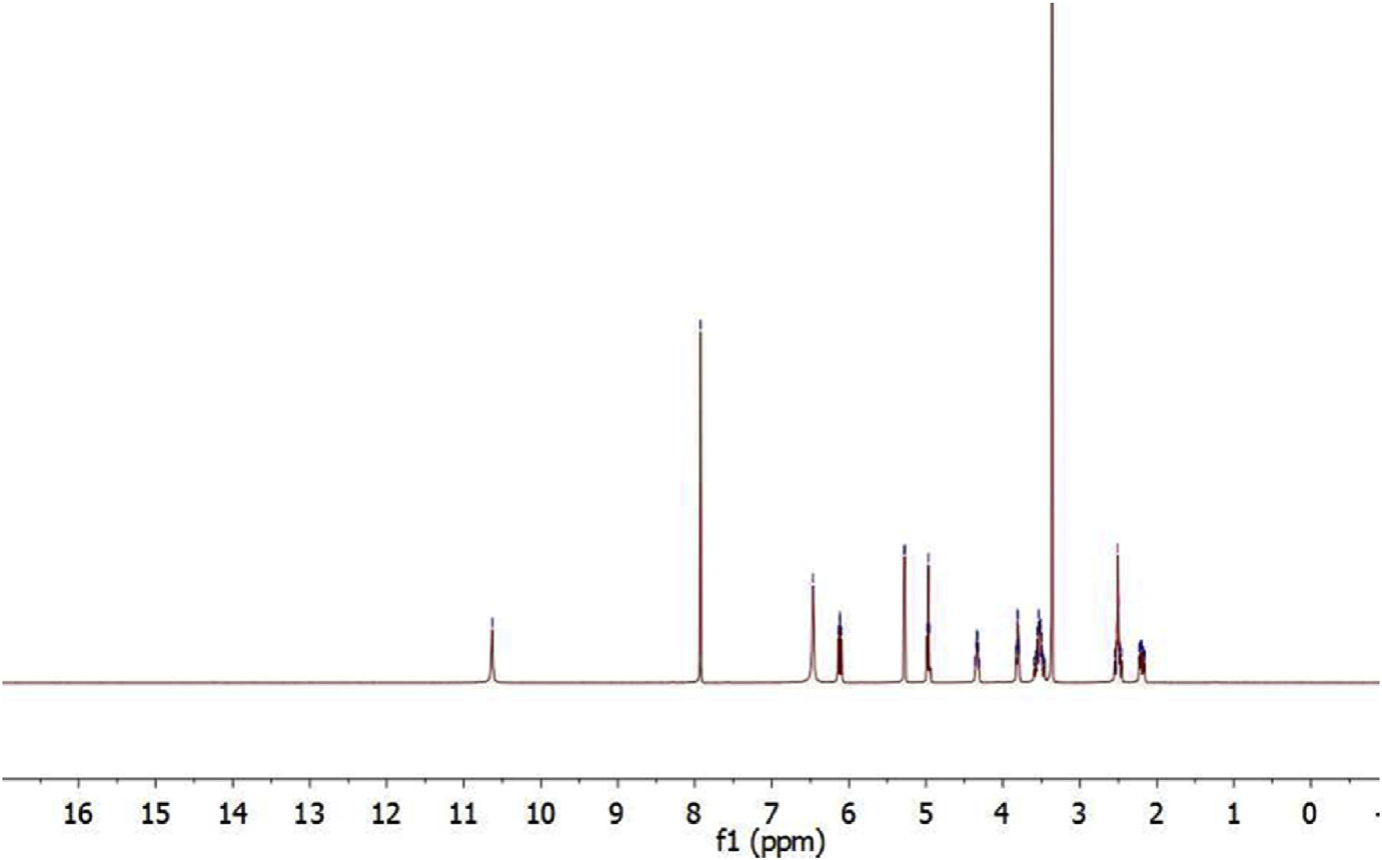
^1^H NMR of guanosine in DMSO.

**Fig. 3:**
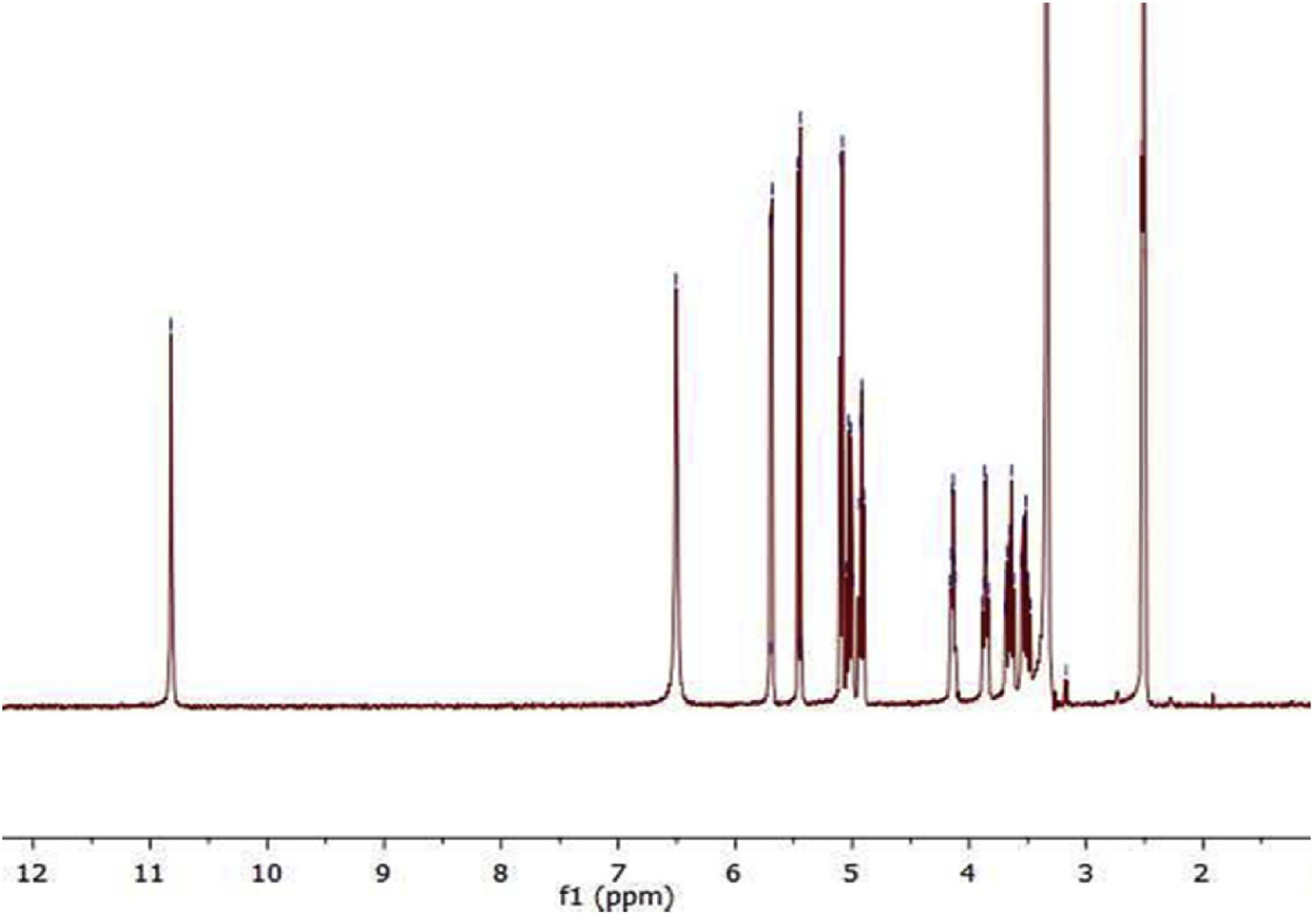
^1^H NMR of 8-bromoguanosine in DMSO.

^1^H NMR (300 MHz DMSO*-d6*, 25 °C) of guanosine spectrum in Fig. 2 showed: 10.63 δ (s, 1H, NH), 7.93 (s,1H, C8), 6.46 (s,2H, NH2), while ^1^HNMR spectrum of 8-bromoguanosine in Fig. 3 displayed: δ 10.82 (s, 1H, NH), 6.55 (s, 2H, NH2). Disappearing the signal that belongs to (1H, C8) in Fig. 3 confirms successful preparation of 8-bromoguanosine.

### 2.7 Synthesis of polymer Ag(I): mixture nucleosides cytidine and 8-bromo guanosine

The one-dimensional (1D) polymer of Ag(I):cytidine and 8-bromoguanosine was prepared with stoichiometry 1:1:1 of Ag(I):cytidine:8-bromoguanosine as followed: The solution of 8-bromoguanosine (0.00615 gm, 16.98 μmol in 200 μL H_2_O) was added to cytidine solution (0.00413 gm, 16.98 μmol in 100 μL DMSO), the mixture was shacked for several minutes then a solution of AgNO_3_ (0.00288 gm, 16.98 μmol in 100 μL H_2_O) was added, then the mixture was shacked quickly and left in a dark place at room temperature. After 30 min colourless viscose solution was formed, the viscosity of the sample was increased with the time. After four days of the preparation, the sample be more viscous, but it was not stable to inversion test to indicate the stability, and this confirms that the sample was not gel. 1.5 μL of the sample was drop-casted on a silicon chip and left to dry by air prior to scan by AFM.

## 3 Results and discussion

### 3.1 Preparation and characterization of Ag(I)-mixture nucleosides, cytidine and 8-bromoguanosine

**Fig. 4:**
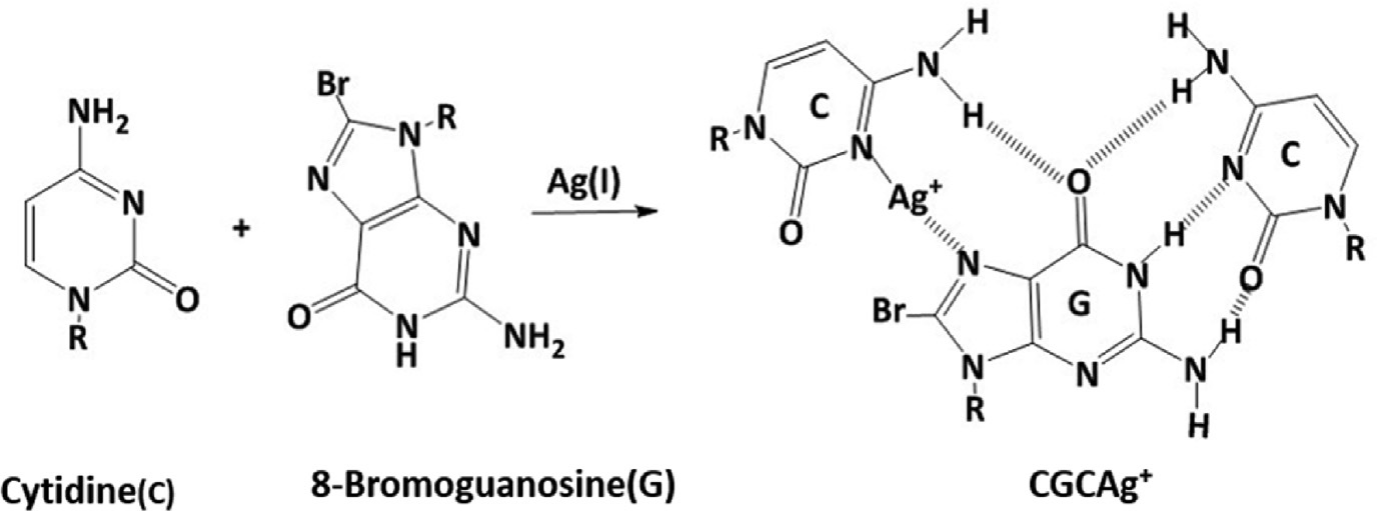
Reaction scheme of cytidine (C) and 8-bromoguanosine (G) in the presence of Ag(I) ions for forming one dimensional polymer of triplex CGCAg^+^.

The reaction of Ag(I) ions with equimolar equivalents mixture nucleosides, cytidine and 8-bromoguanosine, in aqueous solution leads to form triplex pyrimidine motif (Y), CGCAg^+^, as shown in Fig. 4. The coordination of Ag(I) ion in the triplex CGC structure occurs via N3 atom of the cytidine molecular [24, 25], in fact, the presence of Ag(I) ions play fundamental role in the stability of the this structure [24, 26]. Protonation N3 atom in natural medium in cytidine molecular is necessary for complementarity binding with 8-bromoguanosine via Hoogsteen hydrogen bonding in CGC structure [27], this process provides appropriate site for coordinating Ag(I) ion to the cytidine molecular, and gives a rise to increase the stability of this structure, such protonation does not occur in the triplex GCG, and this confirms that the motif of the triplex structure of this fibrous polymer is CGC rather than GCG motif. The structure of the triplex Y motif in this polymer consists of two pyrimidine molecules (cytidine) and one purine molecular (8-bromoguanosine) to form CGCAg^+^, purine and pyrimidine molecules are clustered in the same strand by assisting of strand-switch mechanism.

**Fig. 5:**
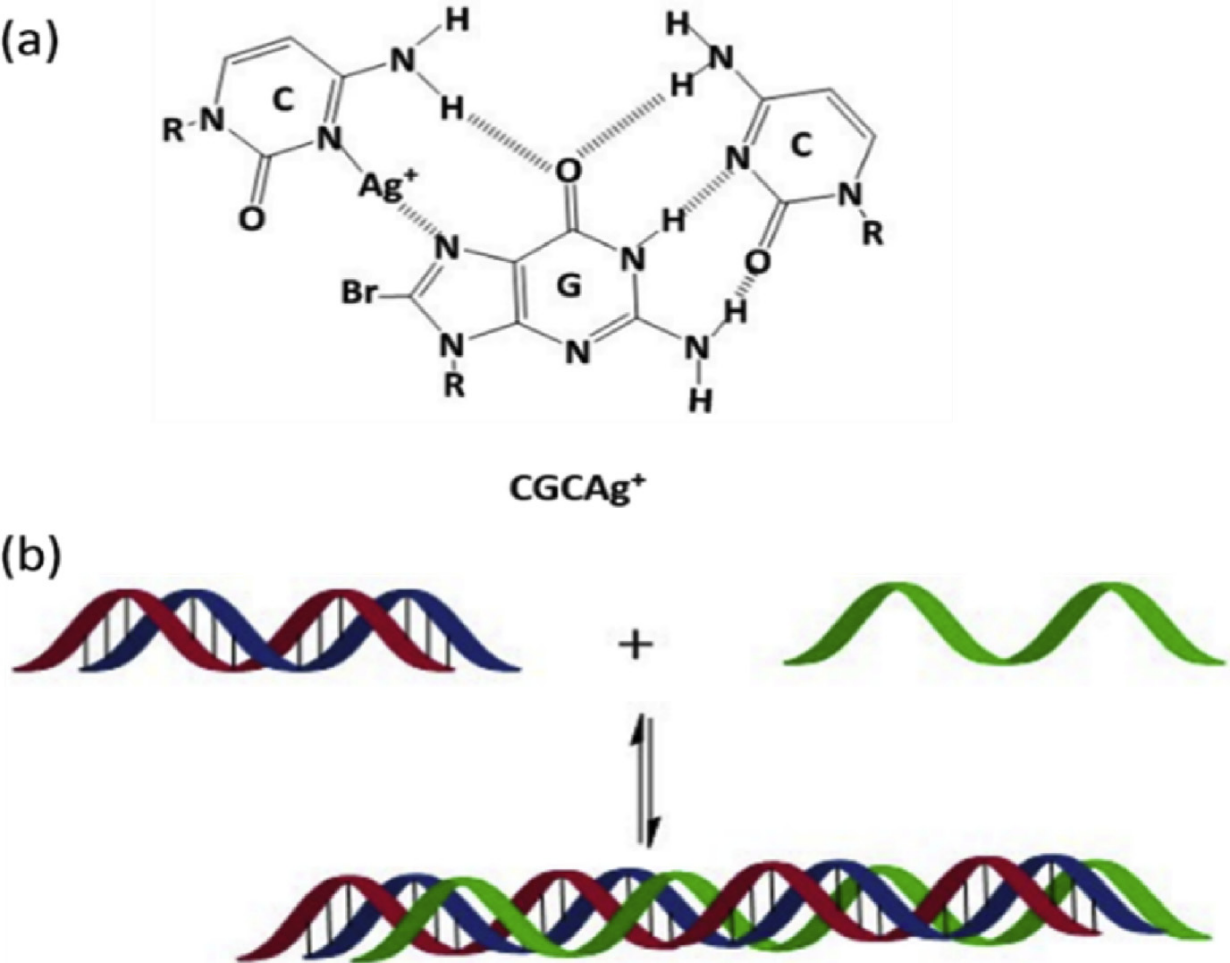
Shows: (a) Triplexes structure of pyrimidine parallel motif CGC bases with Ag(I) ion. (b) Triplexes block of CGCAg+.displaying that the location of the third strand (green colour) is in the major groove of the double helix (red & blue colures) that is built via Hoogsteen hydrogen bonding.

Building triplex block of CGCAg^+^ needs that the position of the third strand must be in the major groove of the double helix that is formed by Hoogsteen hydrogen bonding [27]. Triplexes structures of Y motif can be found in both RNA and DNA by forming intramolecular triplexes. Fig. 5 (a) showing the Triplet structure of parallel motif CGC bases with Ag(I) ion, while Fig. 5 (b), modified from Sugimoto [28], displays building the triplex block, the green strand represents the third strand that be in the major groove of the double strands. Substitution C8 in guanosine [13, 15, 29, 30] molecular in Y motif can increase the stability of the triplex structure in addition to the stability that produces from the presence of Ag(I) ions and the influence of Hoogsteen hydrogen bonding in this structure. Most triplexes structures that concern RNA are synthesised regarding functionally RNAs such as ribosomal RNAs [31], telomerase RNAs [32], and long noncoding RNAs [33, 34]. In addition, these materials played a great role in molecular biology [35] and nanotechnology applications [36, 37].

### 3.2 Atomic force microscopy (AFM) for surface characterization

**Fig. 6:**
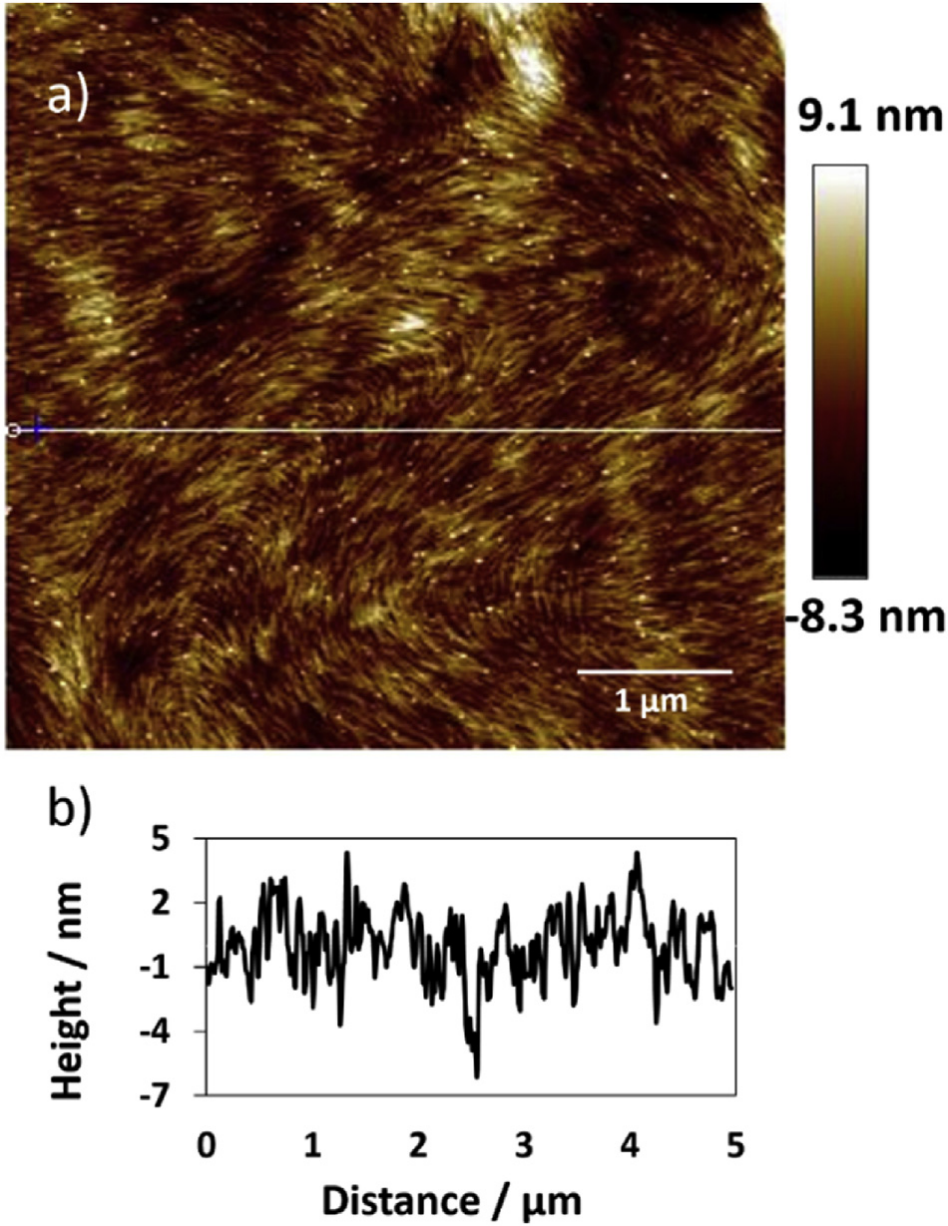
(a)Tapping mode height AFM image (scan area 5 × 5 μm^2^) of the Ag (I): mixture nucleosides polymer, shows the height of the polymer was in the range of 2–3 nm with very few peaks with height up to 5 nm. (b) The profile associated with the horizontal white line along image (a) showing the height of the fibres. The white dots in image (a) refer to the loops that formed by Watson-Crick and Hoogsteen hydrogen bonding.

**Fig. 7:**
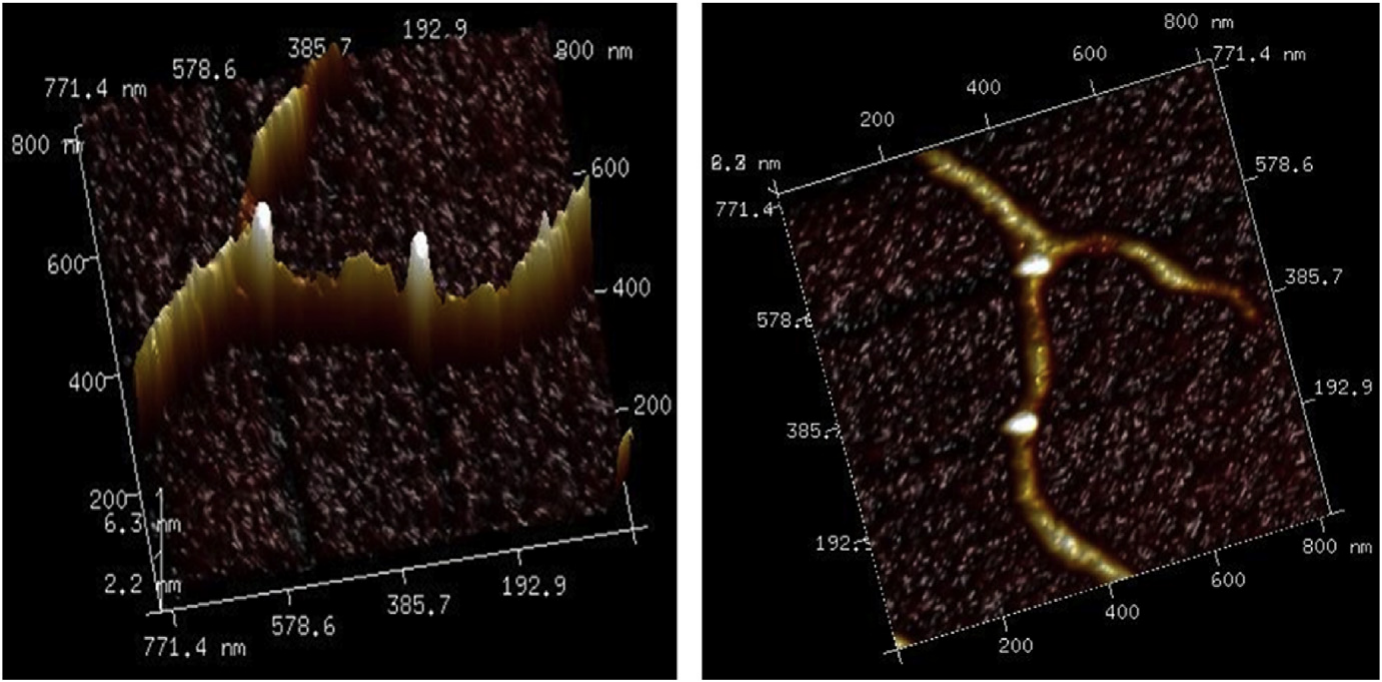
Shows AFM images of the single polymer in 3D view to display the loops that formed in the triplex polymer.

**Fig. 8:**
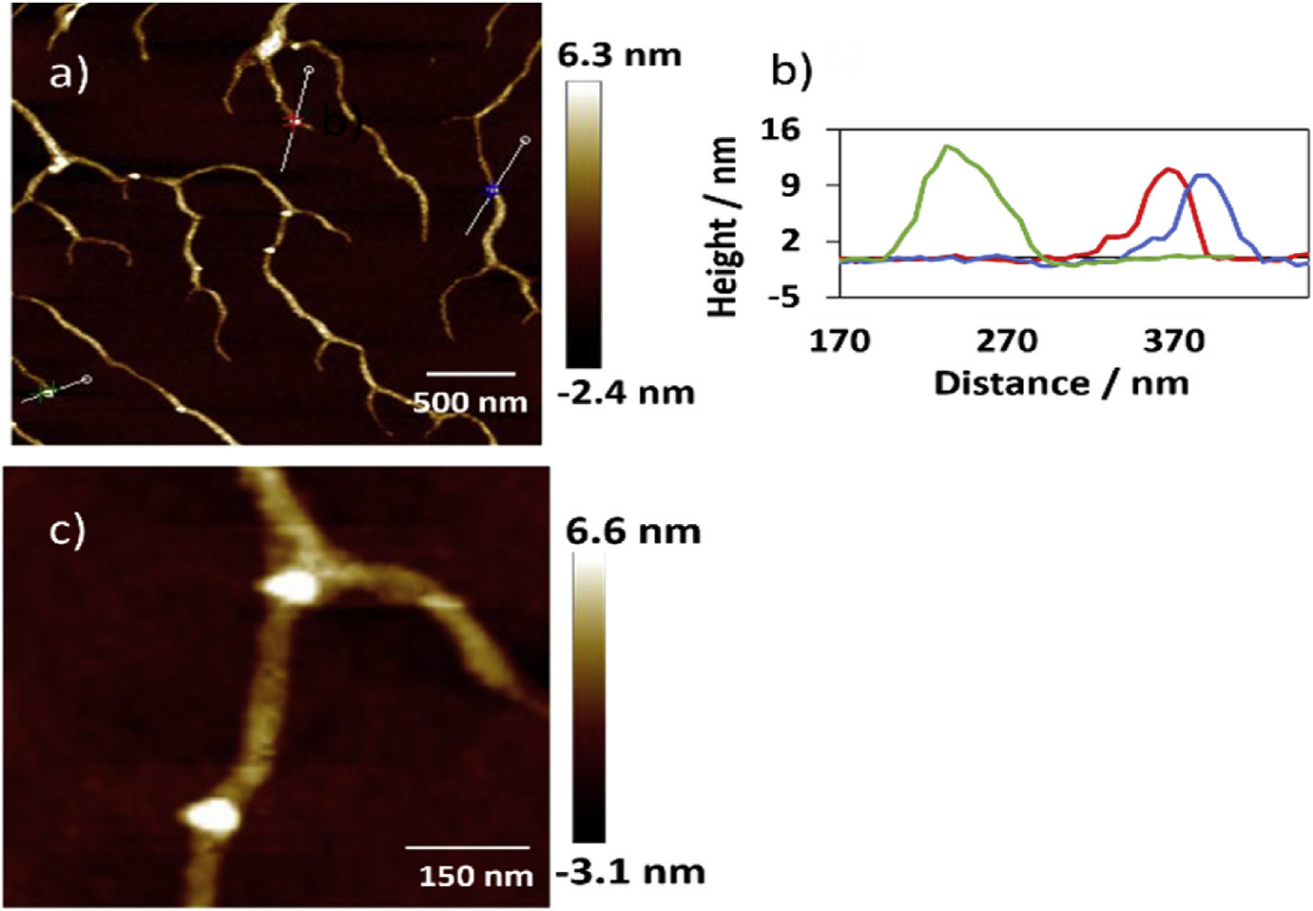
Shows: (a) the height AFM image of the polymer with scale bar 500 nm, and (b) is the profile of the of the blue, red, and green lines across image (a) demonstrating the height of the loops that formed in the polymer, (c) small area of image (a) with scale bar 150 nm.

The AFM technique was used to address the morphology of the polymer. Examination the dried polymer revealed formation of nanofibres extending many microns in length with a height in range of 2–3 nm, some few fibres revealed with height up to 4 nm. Fig. 6 displays tapping mode height AFM image that obtained by scanning area 5 × 5 μm^2^. Many loops can be seen in image (a), Fig. 6 that formed as a consequent of binding of these complementary nucleosides. Fig. 7 displays more AFM images with 3D view for those loops that formed in the triplex polymer. Statistical analysis was carried out to investigate the height of the loops that can be seen in a single polymer in AFM image (a) in Fig. 8. The data displayed that the height values were in the range of 10–14 nm, as shown in Fig. 8 (b) which represents the profile of the three sloping lines in the image (a). Image (c) in Fig. 8 is a small area of image (a) with scale bar 150 nm. These findings indicate that the complementary binding cytidine and 8-bromoguanosine, that formed triplexes structures of parallel pyrimidine CGCAg^+^, can self-assemble to form nanofibres. Self-assembling of nucleosides was seen for guanosine and its derivatives. Different architectures structures such as cyclic [38], lamellae [39], fibres [40], micelles [41], and films [42] have been reported for self-assembling of some complementary nucleobases and their derivatives. However, this is the first report that presents a simple and direct way to prepare nanofibers by self-assembling complementary nucleosides cytidine & 8-bromoguanosine.

### 3.3 Surface texture parameters analysis: waviness, and roughness statistical values of single polymer

**Fig. 9:**
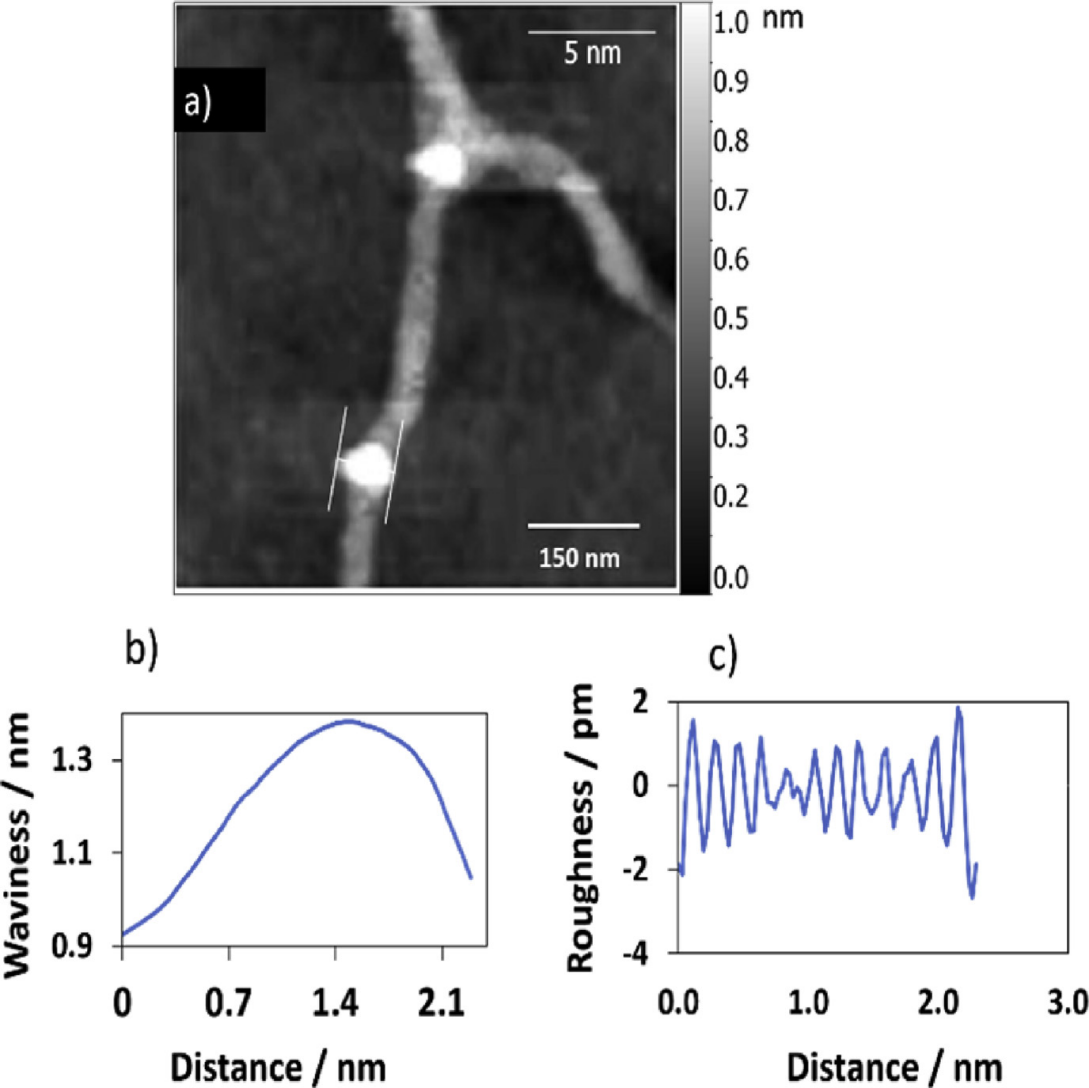
(a) Tipping mode height AFM image with scale bar 150 nm of Ag(I): mixture cytidine and 8-bromoguanosine, the image was analysed by Gwyddion software program with scale 5 nm. The vertical bar that displayed on the right side of the AFM image is corresponding to the highest point (upper limit of the bar) and lowest points (lower point of the bar) that measured in the image in nanometre, respectively. The profiles of surface texture: waviness (b), and roughness (c) are associated with the sloping white lines in image (a).

**Fig. 10:**
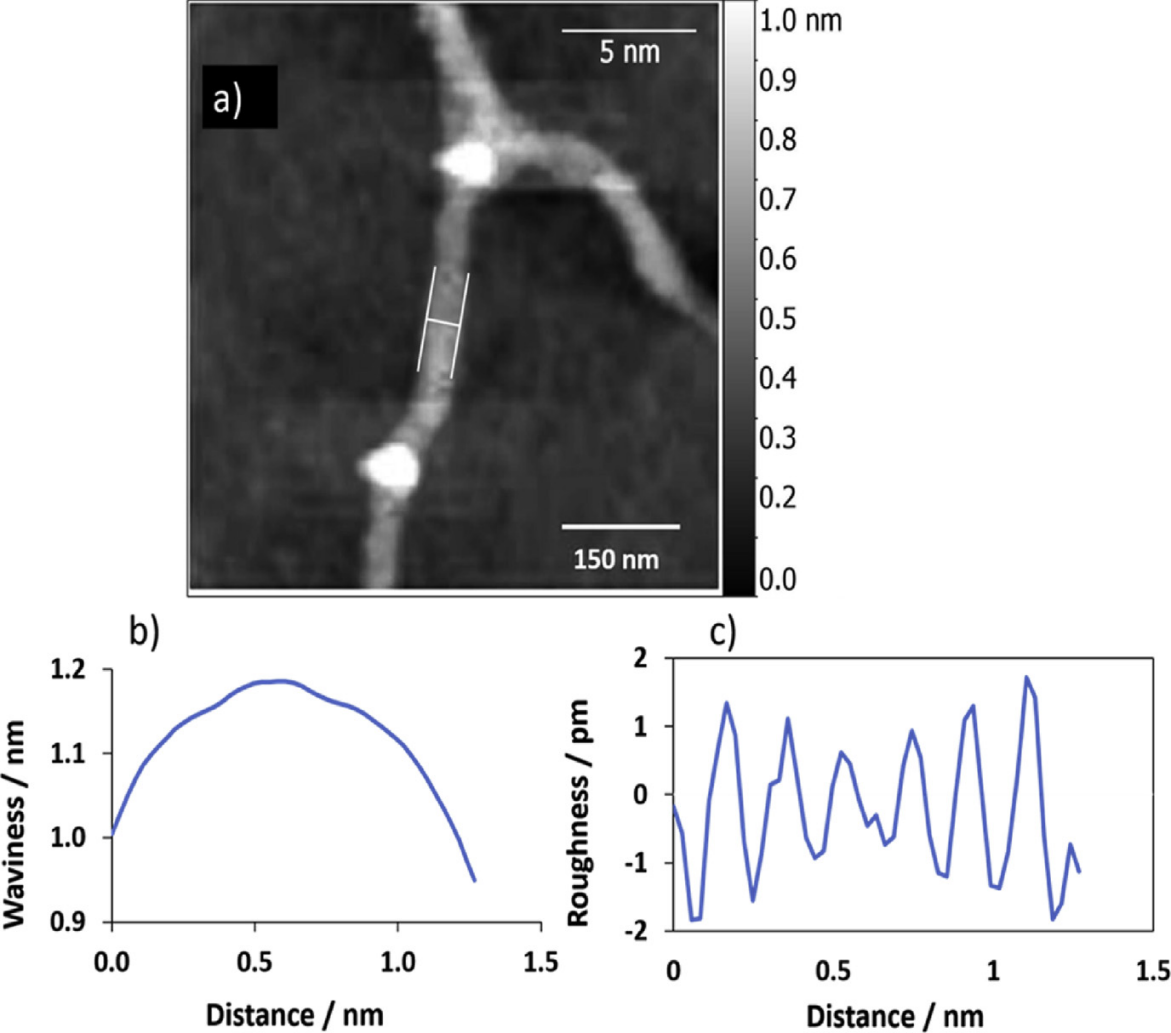
Displays surface roughness parameters of the flat area of the single polymer of AFM image (a). (b) & (c) are the waviness and roughness profiles for the cross section in image (a). The scale bar was 5 nm.

Surface texture analysis is very useful to understand the nature of the material, and it helps in the development of many material components. The surface sample parameters of waviness and roughness of the single polymer were carried out for the AFM image with scanning area 5 × 5 μm^2^ by using Gwyddion software program, the data are presented in Fig. 9 (for the loops) and Fig. 10 for the flat part of the single polymer. The data of Fig. 9 shows that the root mean square roughness (Rq) was 0.9 pm, and the roughness average was 0.7 pm. The Rq parameter, which is related to the standard division of the height distribution of the sample surface, has a special importance as it is more accurate than the average of height arithmetic (Ra), it calls also average roughness, owing to the high value of the deviation from the main line [43, 44]. Eq. (1) can be defined the Rq as:

(1) $$RMS=R_{q}=\sqrt{\frac{1}{L}\int_{0}^{L}Z^{2}\partial x}\approx \sqrt{\frac{1}{n}\sum_{i}^{n}Z_{i}^{2}}$$

RMS is the root mean square average deviation of the roughness, it is known as Rq, also. L and Z (x) are the length of the profile and the function of height profile, respectively. The data demonstrated that the root mean square waviness (Wq) was 147.3 pm, while the waviness average (Wa) was 128.9 pm. The height of the waviness is normally higher than the average roughness height by three times [43]. Tables 1 and 2 summarised the data of Figs. 9 and 10, respectively.

**Table 1:** Statistical parameter values of the height distribution of AFM image in Fig. 9 (scanning area of 5 × 5 μm^2^) for the 1D polymer Ag(I): mixture of nucleosides.

Parameter	Symbol	Value
Amplitude		
Roughness average	Ra	0.7 pm
Root mean square roughness	Rq	0.9 pm
Maximum height of the roughness	Rt	4.5 pm
Maximum roughness valley depth	Rv	2.7 pm
Maximum roughness peak height	Rp	1.9 pm
Average maximum height of the roughness	Rtm	2.7 pm
Average maximum roughness valley depth	Rvm	1.5 pm
Average maximum roughness peak height	Rpm	1.2 pm
Average third highest peak to third lowest valley height	R3z	2.7 pm
Average third highest peak to third lowest valley height	R3z ISO	0.9 pm
Average maximum height of the profile	Rz	2.7 pm
Average maximum height of the roughness	Rz ISO	2.7 pm
Skewness	Rsk	-0.256
Kurtosis	Rku	2.890
Waviness average	Wa	128.9 pm
Root mean square waviness	Wq	147.3 pm
Waviness maximum height	Wy = Wmax	1382.2 pm
Maximum height of the profile	Pt	1381.5 pm
**Spatial**		
Average wavelength of the profile	λa	0.20 nm
Root mean square (RMS) wavelength of the profile	λq	0.20 nm
**Hybrid**		
Average absolute slope	Δa	23.46 10ˆ-3
Root mean square (RMS) slope	Δq	28.89 10ˆ-3
Length	L	2.32 nm
Developed profile length	L0	2.32 nm
Profile length ratio	Ir	1.077

**Table 2:** Statistical parameter values of the height distribution of AFM image in Fig. 10 (scanning area of 5 × 5 μm^2^) of the 1D polymer Ag(I): mixture of nucleosides.

Parameter	Symbol	Value
Amplitude		
Roughness average	Ra	0.8 pm
Root mean square roughness	Rq	0.9 pm
Maximum height of the roughness	Rt	3.6 pm
Maximum roughness valley depth	Rv	1.8 pm
Maximum roughness peak height	Rp	1.7 pm
Average maximum height of the roughness	Rtm	2.5 pm
Average maximum roughness valley depth	Rvm	1.4 pm
Average maximum roughness peak height	Rpm	1.1 pm
Average third highest peak to third lowest valley height	R3z	2.9 pm
Average third highest peak to third lowest valley height	R3z ISO	0.0 pm
Average maximum height of the profile	Rz	2.6 pm
Average maximum height of the roughness	Rz ISO	2.5 pm
Skewness	Rsk	0.166
Kurtosis	Rku	2.217
Waviness average	Wa	50.6 pm
Root mean square waviness	Wq	62.0 pm
Waviness maximum height	Wy = Wmax	1185.5 pm
Maximum height of the profile	Pt	1185.6 pm
**Spatial**		
Average wavelength of the profile	λa	0.20 nm
Root mean square (RMS) wavelength of the profile	λq	0.19 nm
**Hybrid**		
Average absolute slope	Δa	25.31 10ˆ-3
Root mean square (RMS) slope	Δq	31.39 10ˆ-3
Length	L	1.30 nm
Developed profile length	L0	1.30 nm
Profile length ratio	Ir	1.077

### 3.4 Kurtosis parameter

Kurtosis parameter describes the height distribution of the surface [45], the data in Table 1 shows that the value of Kurtosis was 2.89, and this confirms that the distribution curve has low height peaks and the morphology of the loop in the AFM image is valley rather than platykurtic valley, and this observation is very important as it gives a good estimation about the nature of the surface roughness of the polymer. Low peaks are expected to be found in the distribution curve when the value of this parameter <3 and vice versa [45].

### 3.5 Surface polymer analysis using surface roughness parameters

**Fig. 11:**
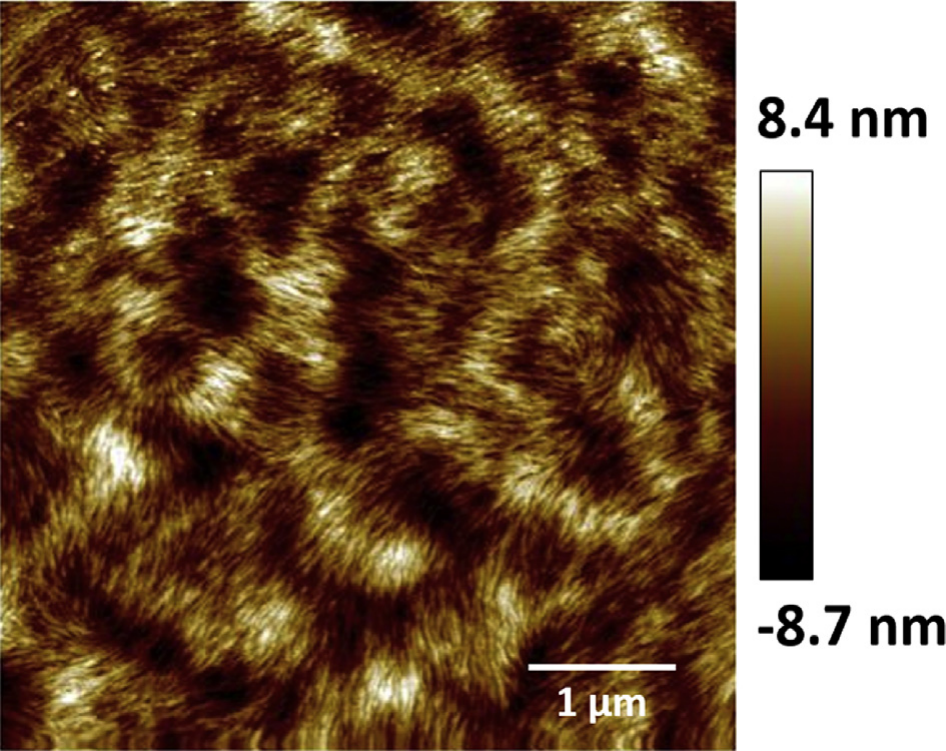
Displays tapping mode height AFM image with scale bar 1 μm (scan area 5 × 5 μm^2^) for the polymer of Ag(I): mixture nucleosides. This image was analysed using Gwyddion software program to obtain surface parameters.

**Fig. 12:**
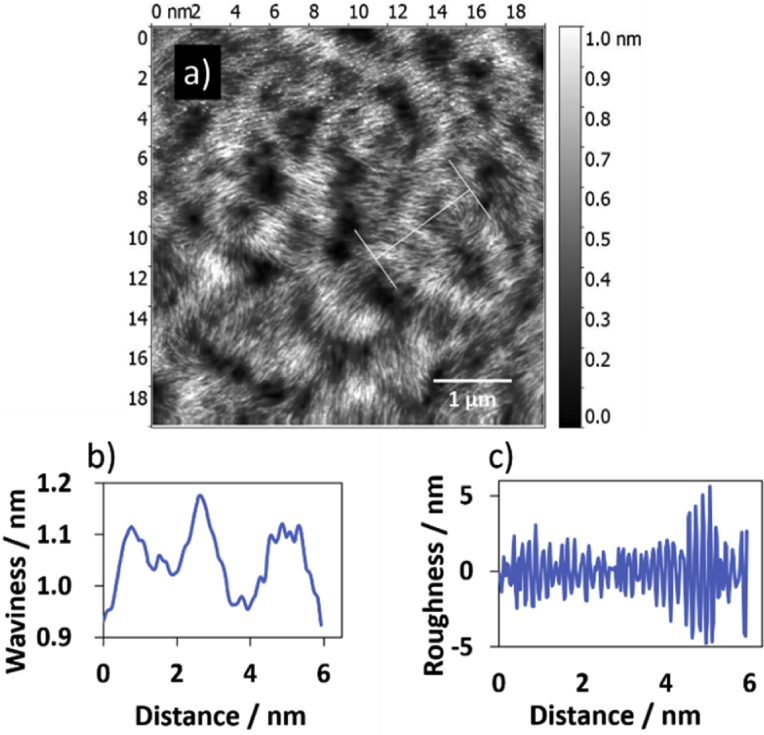
Shows (a) surface analysis of AFM image (a) (scan area 5 × 5 μm^2^). The vertical bar that displayed on the right side of the AFM image is corresponding to the highest point (upper limit of the bar) and lowest points (lower point of the bar) that measured in the image in nanometre, respectively, by using Gwyddion software program. The profiles of surface texture: waviness (b), and roughness (c) are the profile of the sloping white line in the AFM image (a). Selected line: (11.39, 11.64) to (16.19, 8.15) nm.

Gwyddion software program was used to obtain surface roughness parameters by analysis the tapping mode height AFM image, Fig. 11, with scale bar 1 μm (scan area 5 × 5 μm^2^) for the polymer of Ag(I): mixture nucleosides. The image was adjusted to greyscale for analysis, as shown in Fig. 12(a). The scale of the chosen line was 5 nm. Table 3 displays the data that obtained for analysis AFM image (a) in Fig. 12. The data shows that the value of the Kurtosis was >3 and this indicates that the distribution curve is platykurtic rather than a valley [45]. On the other hand, the data showed that the value of Ra (1.3) is less than Rq (1.7) by ∼ 30 %, and this confirms that the roughness profile is following Gaussian distribution [46, 47]. For Gaussian surface; Rq and Ra are interchangeable as shown in Eq. (2):

(2) $${\text{R}}_{\text{q}}∼\sqrt{\frac{\text{π}}{2}}\text{Ra}∼1.25\times {\text{R}}_{\text{a}}$$

**Table 3:** Statistical parameter values of the height distribution of AFM image (a) in Fig. 12 in the main text (scanning area of 5 × 5 μm^2^) of the 1D polymer Ag(I): mixture of nucleosides.

Parameter	Symbol	Value
Amplitude		
Roughness average	Ra	1.3 pm
Root mean square roughness	Rq	1.7 pm
Maximum height of the roughness	Rt	10.4 pm
Maximum roughness valley depth	Rv	4.8 pm
Maximum roughness peak height	Rp	5.6 pm
Average maximum height of the roughness	Rtm	6.4 pm
Average maximum roughness valley depth	Rvm	3.1 pm
Average maximum roughness peak height	Rpm	3.3 pm
Average third highest peak to third lowest valley height	R3z	8.7 pm
Average third highest peak to third lowest valley height	R3z ISO	4.6 pm
Average maximum height of the profile	Rz	8.8 pm
Average maximum height of the roughness	Rz ISO	6.4 pm
Skewness	Rsk	-0.072
Kurtosis	Rku	3.865
Waviness average	Wa	49.9 pm
Root mean square waviness	Wq	59.4 pm
Waviness maximum height	Wy = Wmax	1176.2 pm
Maximum height of the profile	Pt	1176.5 pm
**Spatial**		
Average wavelength of the profile	λa	0.16 nm
Root mean square (RMS) wavelength of the profile	λq	0.16 nm
**Hybrid**		
Average absolute slope	Δa	0.05044
Root mean square (RMS) slope	Δq	0.06678
Length	L	5.96 nm
Developed profile length	L0	5.97 nm
Profile length ratio	Ir	1.033

**Fig. 13:**
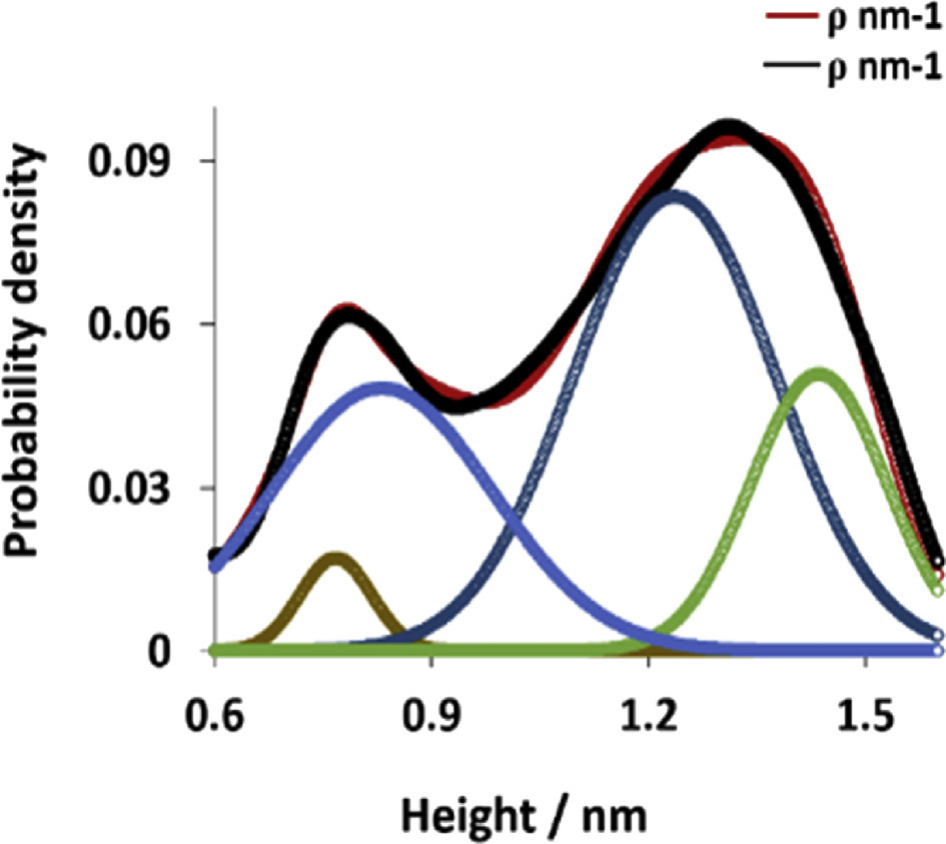
Shows fibre high distribution for the polymer. The black line is a fitted regression model with a sum of four Gaussian functions, while the coloured lines displayed the individual Gaussians.

**Fig. 14:**
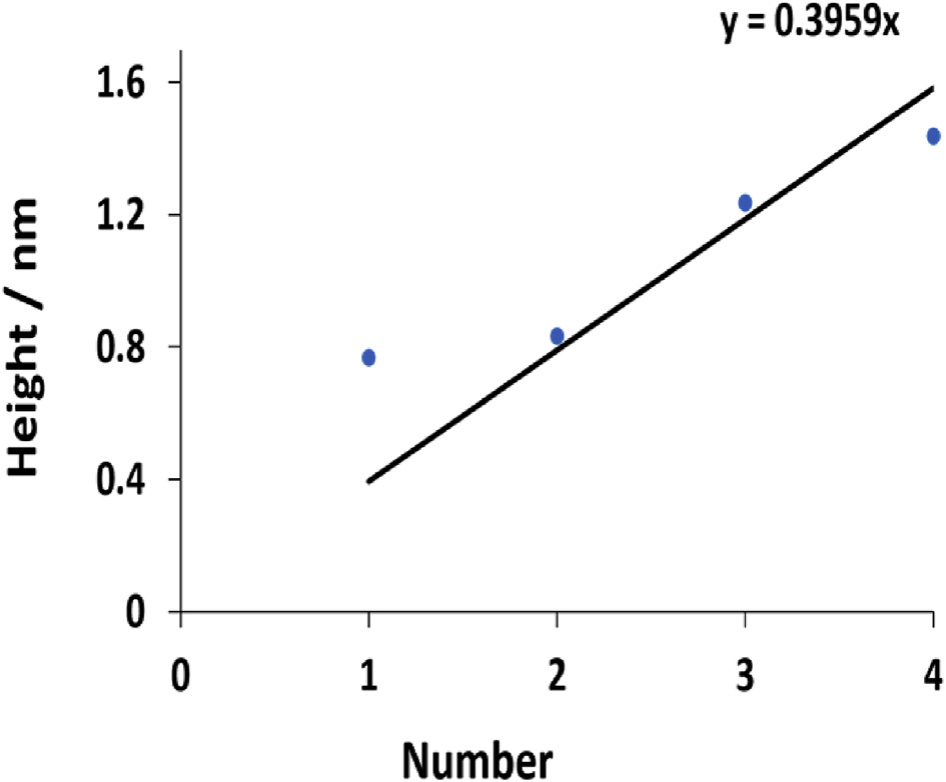
Displays fibre diameter (size distribution) estimated from the peaks heights in Fig. 13. The data shows that the diameter was ∼0.4 nm.

The height distribution of the AFM image (a) in Fig. 12 is fitted to a sum of Gaussian functions to obtain the probability density of the fibres, as shown in Fig. 13, and the straight line in Fig. 14 is fitted through the peak (height) values to get an estimate of ∼0.4 nm diameter for the fibres.

### 3.6 Transmission electron microscopy (TEM)

**Fig. 15:**
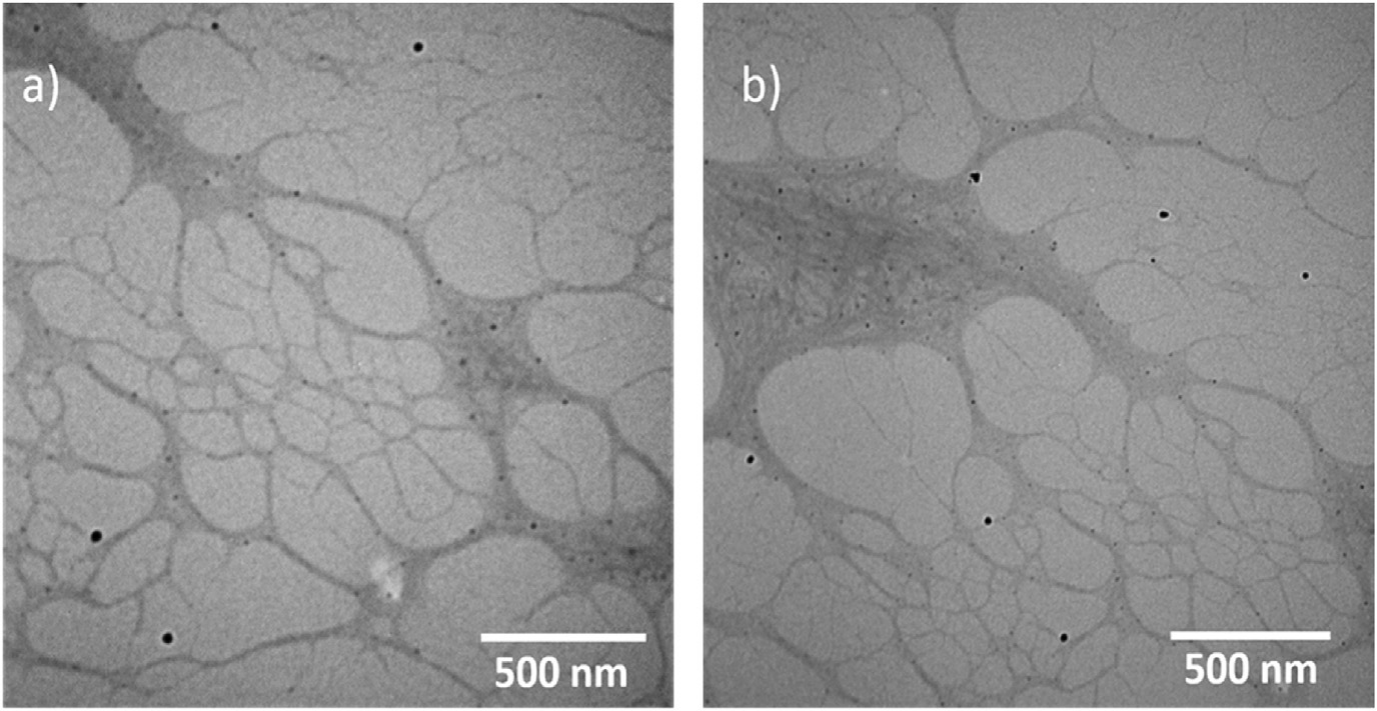
Shows TEM images of the 1D polymer of Ag(I): mixture nucleosides, the scale bar was 500 nm in both images (a) and (b) while the magnification was 46000x and 34000x, respectively.

**Fig. 16:**
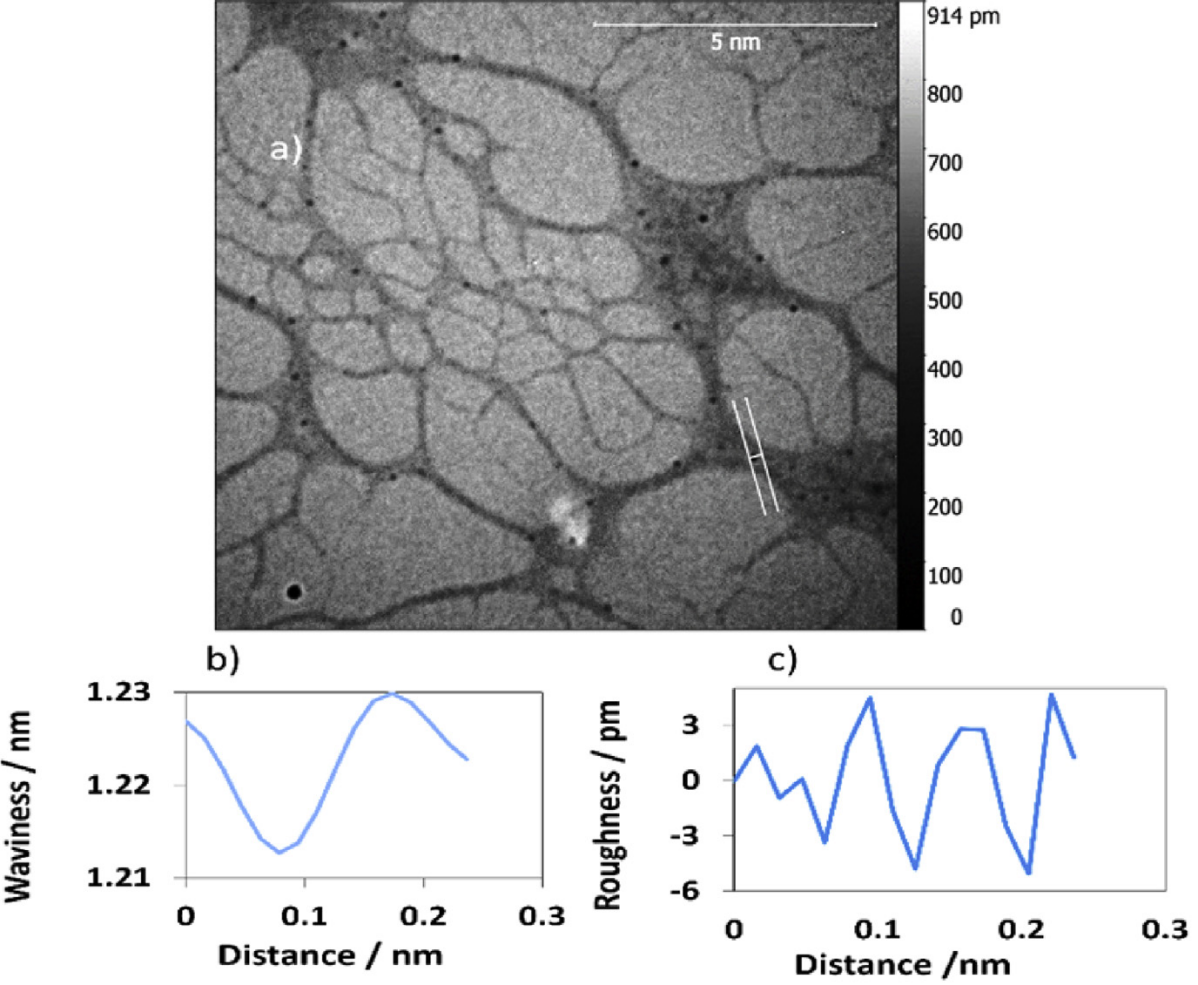
(a) TEM image analysis,(b) & (c) are the waviness and roughness profiles for the cross section a long image (a). The scale bar was 5 nm.

**Fig. 17:**
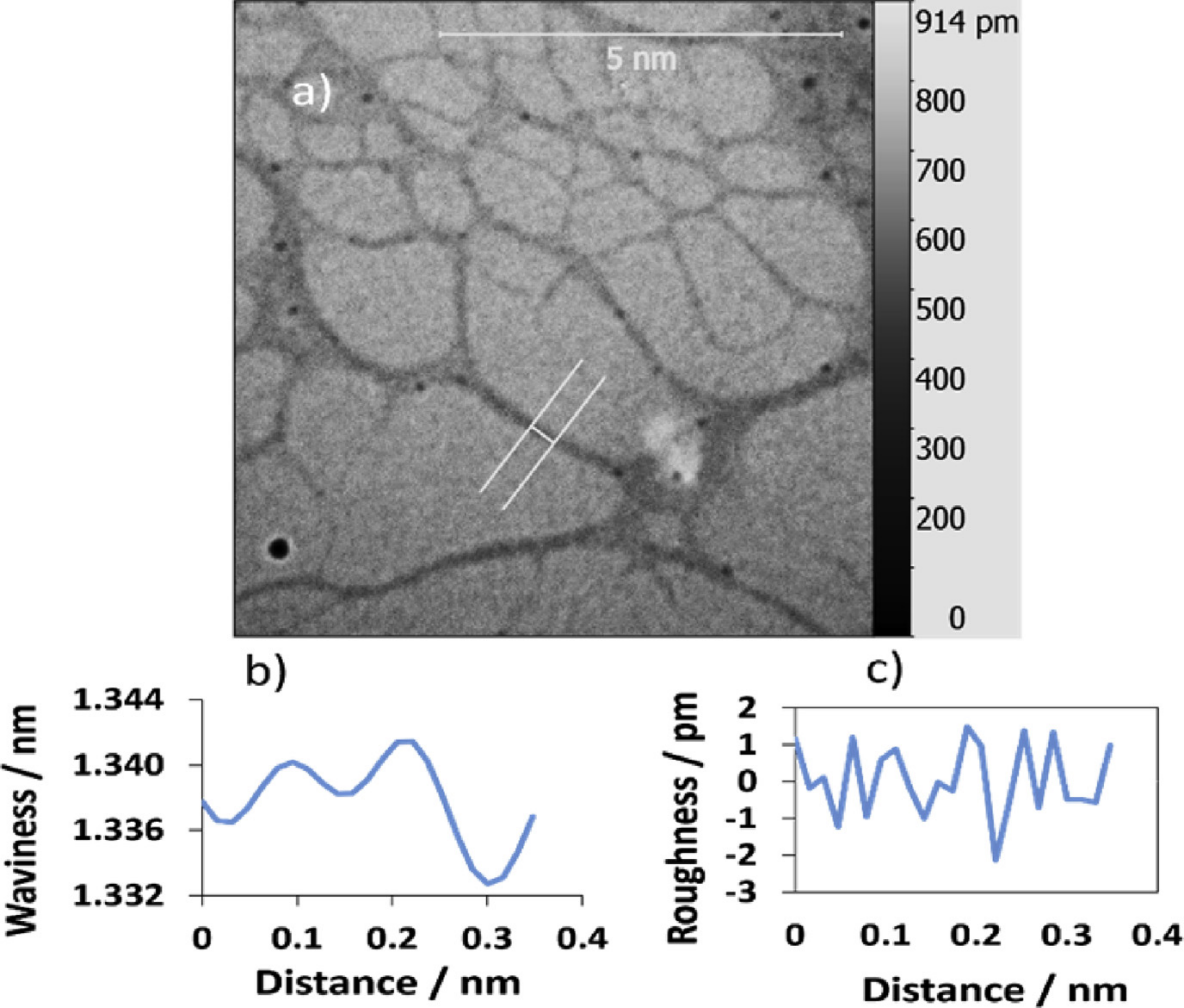
Shows: (a) TEM image surface analysis, (b) & (c) are waviness & roughness profiles of the flat area of the single polymer in image (a). The scale bar was 5 nm.

TEM technique was used to investigate the morphology of the polymer. Carbon coated copper grid was used as a substrate to prepare the sample. 1.5 μL of the sample was drop-casted onto the substrate, left to dry by air prior to imaging. The inspection revealed the formation of a very long, entangled fibres, as shown in Fig. 15. The surface of the single polymer was investigated for the TEM images by using Gwyddion software program to obtain parameters of waviness and roughness, Figs. 16 and 17 display the data for the loops and for the flat part of the single polymer, respectively. Table 4 presents the data of Fig. 16. The data shows that the root mean square roughness (Rq) was 1.0 pm, and the roughness average was 0.8 pm. In addition, the value of Kurtosis (Rku) was 2.33. The data of TEM images were in a good agreement with that of AFM images and this confirms the accurate measurements for analysis the surface texture of the triplex polymer CGCAg^+^.

**Table 4:** Statistical parameter values of the height distribution of TEM image in Fig. 16 in the main text (scanning area of 5 × 5 μm^2^) of the 1D polymer Ag(I): mixture of nucleosides.

Parameter	Symbol	Value
Amplitude		
Roughness average	Ra	0.8 pm
Root mean square roughness	Rq	1 pm
Maximum height of the roughness	Rt	3.6 pm
Maximum roughness valley depth	Rv	2.1 pm
Maximum roughness peak height	Rp	1.5 pm
Average maximum height of the roughness	Rtm	1.7 pm
Average maximum roughness valley depth	Rvm	0.7 pm
Average maximum roughness peak height	Rpm	1 pm
Average third highest peak to third lowest valley height	R3z	2.3 pm
Average third highest peak to third lowest valley height	R3z ISO	0.0 pm
Average maximum height of the profile	Rz	2.6 pm
Average maximum height of the roughness	Rz ISO	1.7 pm
Skewness	Rsk	-0.196
Kurtosis	Rku	2.330
Waviness average	Wa	2 pm
Root mean square waviness	Wq	2.5 pm
Waviness maximum height	Wy = Wmax	1341.4 pm
Maximum height of the profile	Pt	1342.4 pm
**Spatial**		
Average wavelength of the profile	λa	0.06 nm
Root mean square (RMS) wavelength of the profile	λq	0.06 nm
**Hybrid**		
Average absolute slope	Δa	0.07958
Root mean square (RMS) slope	Δq	0.09789
Length	L	0.36 nm
Developed profile length	L0	0.37 nm
Profile length ratio	Ir	1.009

## 4 Conclusions

In summary, one dimensional triplex parallel pyrimidine polymer of Y motif based on self-assemble of Ag(I) with mixture complementary bases nucleosides (G & C) was prepared, to the best of our knowledge, this is the first report displays that complimentary nucleosides, cytidine & 8-bromoguanosine, are capable of self-assembling directly to produce nanostructure material with such length and height as shown by AFM measurements where the height of the polymer was in the range of 2–3 nm and the length was many microns. This feature makes this polymer analogous to the duplex DNA [4]. Surface roughness was carried out to indicate the probability density of the fibre. The data displayed that the diameter of the fibre was ∼0.4 nm. Waviness, Roughness, and Kurtosis parameter values for the fibrous structure were also investigated by analysis AFM images and TEM images where the data showed a good agreement.

## Declarations

### Author contribution statement

Lamia al-Mahamad: Conceived and designed the experiments; Performed the experiments; Analyzed and interpreted the data; Contributed reagents, materials, analysis tools or data; Wrote the paper.

### Funding statement

This research did not receive any specific grant from funding agencies in the public, commercial, or not-for-profit sectors.

### Competing interest statement

The authors declare no conflict of interest.

### Additional information

No additional information is available for this paper.
